# Work-Related Psychological Wellbeing of Catholic Priests in Portugal: Cross-Cultural Adaptation of the Francis Burnout Inventory

**DOI:** 10.1007/s10943-025-02275-w

**Published:** 2025-03-04

**Authors:** Janaína Mengal Gomes Fabri, Leslie J. Francis, Ursula McKenna, Liliana Isabel Faria Roldão, Eliane Ramos Pereira, Andrew Village, Sílvia Caldeira

**Affiliations:** 1 Faculty of Nursing, State University of Rio de Janeiro, Rio de Janeiro, Brazil; 2 Fluminense Federal University, Rio de Janeiro, Brazil; 3 Health Sciences Institute, Portuguese Catholic University, Lisbon, Portugal; 4 Centre for Educational Development, Appraisal and Research (CEDAR), The University of Warwick, Coventry, UK; 5 World Religions and Education Research Unit, Bishop Grosseteste University, Lincoln, UK; 6 Portuguese Catholic University, Lisbon, Portugal; 7 Universidad Nacional de Trujillo (UNT), Trujillo, Peru; 8 School of Humanities, York St John University, York, UK; 9 Faculty of Health Sciences and Nursing, Centre for Interdisciplinary Research in Health, Universidade Católica Portuguesa, Lisbon, Portugal

**Keywords:** Balanced affect, Emotional exhaustion, Satisfaction in ministry, Clergy, Wellbeing

## Abstract

The present study was designed to translate the Francis Burnout Inventory Revised into Portuguese and to test this translation among a snowball sample of 266 Catholic priests serving in Portugal (91% diocesan). The data demonstrated: good internal consistency reliability for the two scales proposed by this instrument (negative affect, *α* = .89 and positive affect, *α* = .89); support for the association with a measure of self-compassion; and support for the theory of balanced affect against a measure of thoughts of leaving ministry. The priests were found to display a high level of positive affect that masked a degree of negative affect, with a third of them reporting that fatigue and irritation were part of their daily experience.

## Introduction

The assessment and management of burnout among priests, ministers, and clergy are a matter of both scientific and practical concern at a time when in a number of contexts Churches appear to be confronting decline in membership and consequent decline in vocations for ministry. The assessment of burnout depends on clear conceptualisation of the condition and on appropriate operationalisation of the conceptualisation. Management strategies to deal with burnout need also to be closely aligned with the conceptualisation of the condition.

### Maslach Burnout Inventory

Within the caring professions the best established conceptualisation and measurement of burnout is that proposed by the Maslach Burnout Inventory (Maslach & Jackson, 1986). Implicit within this conceptualisation is a three-stage progression from workplace emotional exhaustion, to depersonalisation of clients, to a growing absence of a sense of personal accomplishment within the workplace. Each of these three components is measured by three separate scales within the Maslach Burnout Inventory. From the 1980s onwards the Maslach Burnout Inventory has been employed in studies among priests, ministers, and clergy, including early studies reported by Crea (1994), Strümpfer and Bands (1996), Rodgerson and Piedmont (1998), Stanton-Rich and Iso-Ahola (1998), and Virginia (1998) and more recent studies reported by Crea and Francis (2015), Adams et al. (2017), Büssing et al. (2017), Vicente-Galindo et al. (2017), Chan and Chen (2019), Dias (2019), Case et al. (2020), Muasa et al. (2021), Malcolm et al. (2022), Proeschold-Bell et al. (2022), and Sanagiotto (2024).

In the 1980s Francis’ research group began to raise questions about the effectiveness of employing the Maslach Burnout Inventory among priest, ministers, chaplains and other clergy. Their initial question concerned the appropriateness of the language employed in the Maslach Burnout Inventory for application within the clerical profession. Priest, ministers, chaplains and other clergy rarely refer to those among whom they minister as clients. To address this concern, Francis’ research group acquired permission from the copyright holders to propose a modified form of the Maslach Burnout Inventory that they tested in a series of studies in the UK among Anglican clergy (Francis & Rutledge, 2000; Francis & Turton, 2004a, 2004b; Randall, 2004, 2007, 2013; Rutledge & Francis, 2004), Catholic priests (Francis, Louden, & Rutledge, 2004; Francis, Turton, & Louden, 2007), and Pentecostal pastors (Kay, 2000). These three revised scales, concerning emotional exhaustion, depersonalisation, and personal accomplishment, worked well, but also raised two further questions about the model of burnout implied by the measure.

The first further question concerned establishing the independence of the three measures and validating the sequential model implied by the progressive stages of burnout. This question encouraged Francis’ research group to revisit classic models of psychological wellbeing that regarded positive affect and negative affect as operating as partially independent systems. In particular, they drew on the notion of balanced affect as proposed by Bradburn (1969). According to this model of balanced affect, warning signs of poor work-related psychological health (or burnout) occur when *high* levels of negative affect coincide with *low* levels of positive affect. According to this model, high levels of positive affect may mitigate the deleterious consequences of high levels of negative affect.

The second further question concerned developing pastoral approaches to address burnout among priest, ministers, chaplains and other clergy, either as preventative or as remedial strategies. The balanced affect approach, by differentiating between positive affect and negative affect, addresses this specific question. While the causes of emotional exhaustion (negative affect) may be difficult to remove, given the complex nature of ministry, the enhancement of satisfaction in ministry (positive affect) may be possible through personal programmes of self-awareness and institutional programmes designed to maximise the effective fit between individual predispositions and organisational structure.

### Francis Burnout Inventory

Against this background, Francis’ research group began to operationalise and test a new measure of burnout designed specifically for the clerical profession, and styled the Francis Burnout Inventory (Francis, Kaldor, et al., 2005). In its original form the Francis Burnout Inventory comprised two 11-item scales of emotional exhaustion in ministry (negative affect) and satisfaction in ministry (positive affect). The Satisfaction in Ministry Scale (SIMS) drew together items expressing personal accomplishment, personal satisfaction, the sense of dealing effectively with people, really understanding and influencing people positively, being appreciated by others, deriving purpose and meaning from ministry, and being glad that they entered ministry. The Scale of Emotional Exhaustion in Ministry (SEEM) drew together items expressing lack of enthusiasm for ministry, frustration, impatience, negativity, cynicism, inflexibility, profound sadness, the sense of being drained and exhausted by the job, and withdrawal from personal engagement with the people among whom ministry is exercised. In their foundation study conducted among 6,680 ministers across three nations (Australia, England, and New Zealand), Francis, Kaldor, et al. (2005) reported an alpha coefficient of .84 for SIMS, and an alpha coefficient of .84 for SEEM.

In order to test the notion of balanced affect as operationalised by the Francis Burnout Inventory, a series of independent studies has been designed to examine the theory that high levels of positive affect can offset the deleterious consequences of high levels of negative affect. These studies have posited an outcome variable (or set of variables) against which the effect of scores recorded on the two scales (SEEM and SIMS) can be regressed. In this strategy attention is given to the additional effect of the interaction term created by the measure of negative affect (SEEM) and the measure of positive affect (SIMS). This interaction term is testing the extent to which the mitigating effects of positive affect on the outcome variables increases with increasing levels of negative affect. A key example of the kind of outcome variable employed in this series of studies concerns the frequency of entertaining thoughts of leaving ministry.

This series of studies include work reported by Francis, Village, et al. (2011) among 744 clergy in The Presbyterian Church USA, Francis, Laycock, and Brewster (2017) among 658 clergy in the Church of England, Francis, Laycock, and Crea (2017) among 155 priests in the Roman Catholic Church in Italy, Francis, Crea, and Laycock (2017) among 95 priests and 61 sisters in the Roman Catholic Church in Italy, Village et al. (2018) among 358 Anglican clergy in the Church of Wales, Francis, Laycock, and Ratter (2019) among 99 Anglican clergy in England, Francis, Crea, and Laycock. (2021) among 287 priests in the Roman Catholic Church in Italy, and Francis, Village, and Haley (2023) among 803 Methodist ministers in Britain. In the course of these studies, attention was drawn to the way in which each of the two 11-item scales (SEEM and SIMS) could be improved by the removal of one item. This resulted in the Francis Burnout Inventory Revised (Francis, Crea, & Laycock, 2021) and with the recommendation that future research should focus on this revised instrument.

While the Francis Burnout Inventory was originally developed for use in English-speaking communities, the Italian translation has now been well tested and employed in a series of studies (Francis & Crea, 2015, 2018, 2021; Francis, Crea & Laycock, 2017, 2021,). More recently a translation has been prepared and tested for use among clergy in Brazil (Fabri, et al., under review).

### Research Question

Against this background, the aim of the present study was to translate the Francis Burnout Inventory Revised into Portuguese and to test the application of this translation among a sample of Catholic priests serving in Portugal in terms of internal consistency reliability, association with an established measure of self-compassion, and prediction of thoughts of leaving ministry.

## Method

### Procedure

Before the study reported in the present paper, recognized procedures had been followed for translating the Francis Burnout Inventory for application in Portugal. The stages adopted were those universally recommended by Beaton et al. (2007) for cross-cultural adaptation and validation, namely: assessment of conceptual and item equivalence; assessment of semantic and idiomatic equivalence; pre-test of the final version; presentation of the translated and adapted version of the instrument to the authors; and content validation. For the present study, data were collected from a snowball sample of 266 Catholic priests serving in all regions of Portugal, including diocesan and religious priests. Data were collected using an anonymous online form hosted on the *LimeSurvey* Platform and sent by email or social media. Data were collected from February 2024 to July 2024.

### Participants

Of the 266 Catholic priests serving in Portugal whose data were analysed in this study, 243 (91.3%) were diocesan priests, and 23 (8.7%) were religious priests residing in the seven regions representing the entire Portuguese territory. The mean age was 52.3 years (SD = 14.6); 170 (64.0%) held postgraduate qualifications (see Table 1).

**Table 1:** Sociodemographic characteristics of the participants

Characteristics	*N*	%
*Status*		
Diocesan priest	243	91.3
Religious priest	23	8.7
*Race*		
Black	18	6.8
Brown	6	2.2
Indigenous	1	0.4
Yellow	3	1.1
White	233	87.6
Unknown	5	1.9
*Education*		
University education	96	36.1
Postgraduate specialization	17	6.4
Master’s	111	41.7
Doctorate	42	15.7
*Years in priesthood*		
1 to 5 years	15	5.7
6 to 10 years	23	8.6
11 to 15 years	32	12.0
16 to 20 years	20	7.5
21 to 25 years	29	10.9
26 to 30 years	20	7.5
More 30 years	68	25.6
Unknown	59	22.2

*N* = 266

### Measures

*Work-related psychological wellbeing* was assessed by the two scales proposed by the Francis Burnout Inventory Revised (FBI-R; Francis, Crea, & Laycock, 2021). This instrument comprises two 10-item scales. The 10-item Scale of Emotional Exhaustion in Ministry (SEEM) assesses negative affect. The 10-item Satisfaction in Ministry Scale (SIMS) assesses positive affect. Each item is rated on a five-point Likert scale: disagree strongly (1), disagree (2), not certain (3), agree (4), and agree strongly (5). Francis, Crea, and Laycock (2021) reported the following Cronbach alphas for the two scales: SEEM, *α* = .78; SIMS, *α* = .84.

*Self-compassion* was assessed by the Self-Compassion Scale proposed by Neff (2003a, 2003b). This instrument comprises 26 items that measure three positive components of self-compassion (self-kindness, common humanity, and mindfulness) and three negative components of self-compassion (self-judgement, isolation, and over-identification). Each item is rated on a five-point Likert scale anchored by the two poles: almost never (1) and almost always (5). Neff (2003b) reported Cronbach’s alpha of .92 for the total scale score.

*Thoughts of leaving ministry* were rated on a four-point scale: never (1), once or twice (2), several times (3), often (4).

### Analysis

The data were analyzed by means of the SPSS software using the frequency, reliability, correlation, and regression routines.

## Results and Discussion

The first step in data analysis examines the psychometric properties of the two scales proposed by the FBI-R (SEEM and SIMS) in terms of the correlations between the individual items and the sum of the other nine items within the scale, and the item endorsement as the sum of the strongly agree and agree responses (see Table 2). The correlations confirm that within each scale each of the ten items correlates above .4 with the sum of the other items in the scale. Within SEEM the item with the highest correlation with the sum of the other items in the scale was ‘I find myself frustrated in my attempts to accomplish tasks important to me’ (*r* = .71). This item captures the experience of frustration at the heart of emotional exhaustion as operationalised by the FBI-R. Within SIMS the item with the highest correlation with the sum of the other items in the scale was ‘I gain a lot of personal satisfaction from fulfilling my ministry roles’ (*r* = .72). This item captures the experience of personal satisfaction at the heart of satisfaction in ministry as operationalised by the FBI-R.

**Table 2:** Francis Burnout Inventory Revised: Scale properties

	*r*	%
*Scale of Emotional Exhaustion in Ministry*		
I feel drained by fulfilling my ministry roles	.61	21
Fatigue and irritation are part of my daily experience	.63	34
I am invaded by sadness I can’t explain	.69	11
I am feeling negative or cynical about the people with whom I work	.64	27
My humour has a cynical and biting tone	.42	12
I find myself spending less and less time with those among whom I minister	.59	26
I have been discouraged by the lack of personal support for me here	.68	29
I find myself frustrated in my attempts to accomplish tasks important to me	.71	17
I am less patient with those among whom I minister than I used to be	.59	26
I am becoming less flexible in my dealings with those among whom I minister	.67	17
*Satisfaction in Ministry Scale*		
I have accomplished many worthwhile things in my current ministry	.42	92
I gain a lot of personal satisfaction from working with people in my current ministry	.60	89
I deal very effectively with the problems of the people in my current ministry	.50	76
I feel very positive about my current ministry	.68	81
I feel that my pastoral ministry has a positive influence on people’s lives	.63	93
I feel that my teaching ministry has a positive influence on people’s faith	.59	88
I feel that my ministry is really appreciated by people	.71	85
I am really glad that I entered the ministry	.70	89
The ministry here gives real purpose and meaning to my life	.69	87
I gain a lot of personal satisfaction from fulfilling my ministry roles	.72	81

*r* = correlation between individual items and the sum of the remaining items. % = item endorsement, combining the agree and agree strongly responses

In terms of emotional exhaustion in ministry, more than a quarter of the Catholic priests who participated in the study endorsed five items within this scale, indicating that: fatigue and irritation were part of their daily experience (34%); they have been discouraged by the lack of personal support for them in their ministry (29%); they are feeling negative or cynical about the people with whom they work (27%); they find themselves spending less and less time with those among whom they minister (26%); and they are less patient with those among whom they minister than they used to be (26%). At least one in six of the Catholic priests endorsed a further three items in the scale, indicating that: they feel drained by fulfilling their ministry roles (21%); they find themselves frustrated in their attempts to accomplish tasks important to them (17%); and they are becoming less flexible in their dealings with those among whom they minister (17%). At least one in ten of the Catholic priests endorsed the remaining two items in the scale, indicating that: their humour has a cynical and biting tone (12%); and they are invaded by a sadness that they cannot explain (11%).

In terms of satisfaction in ministry, at least four in every five of the Catholic priests who participated in the study endorsed nine of the ten items within this scale, indicating that: they feel that their pastoral ministry has a positive influence on people’s lives (93%); they have accomplished many worthwhile things in their current ministry (92%); they gain a lot of personal satisfaction from working with people (89%); they are really glad that they entered the ministry (89%); they feel that their teaching ministry has a positive influence on people’s faith (88%); their ministry gives real purpose and meaning to their life (87%); they feel that their ministry is really appreciated by people (85%); they gain a lot of personal satisfaction from fulfilling their ministry roles (81%); and they feel very positive about their current ministry (81%). The remaining item was endorsed by three quarters of the Catholic priests, indicating that they deal very effectively with the problems of people in their current ministry (76%).

The second step in data analysis summarises the scale properties of the three continuous scale scores employed in the present analyses (the two scales of the Francis Burnout Inventory Revised and the Self-Compassion Scale), in terms of means, standard deviations, and internal consistency reliability (Cronbach, 1951). The alpha coefficients demonstrate the good internal consistency reliability of all three measures (see Table 3).

**Table 3:** Scale properties of the Francis Burnout Inventory Revised and Self-Compassion Scale

	Alpha	*N* items	Mean	*SD*
Scale of Emotional Exhaustion in Ministry	.89	10	24.01	7.52
Satisfaction in Ministry Scale	.89	10	41.22	5.15
Self-Compassion Scale	.92	26	86.75	10.78

The third step in data analysis explores the responses to the question concerning thoughts of leaving ministry, the index employed as an independent measure of burnout. While three fifths of the priests had never entertained thoughts of leaving ministry (59%), the other two fifths had done so, with 17% having entertained such thoughts on more than a couple of occasions (see Table 4).

**Table 4:** An independent measure of burnout

	%
*Thoughts of leaving ministry*	
Never	59
Once or twice	25
Several times	11
Often	6

**Fig. 1:**
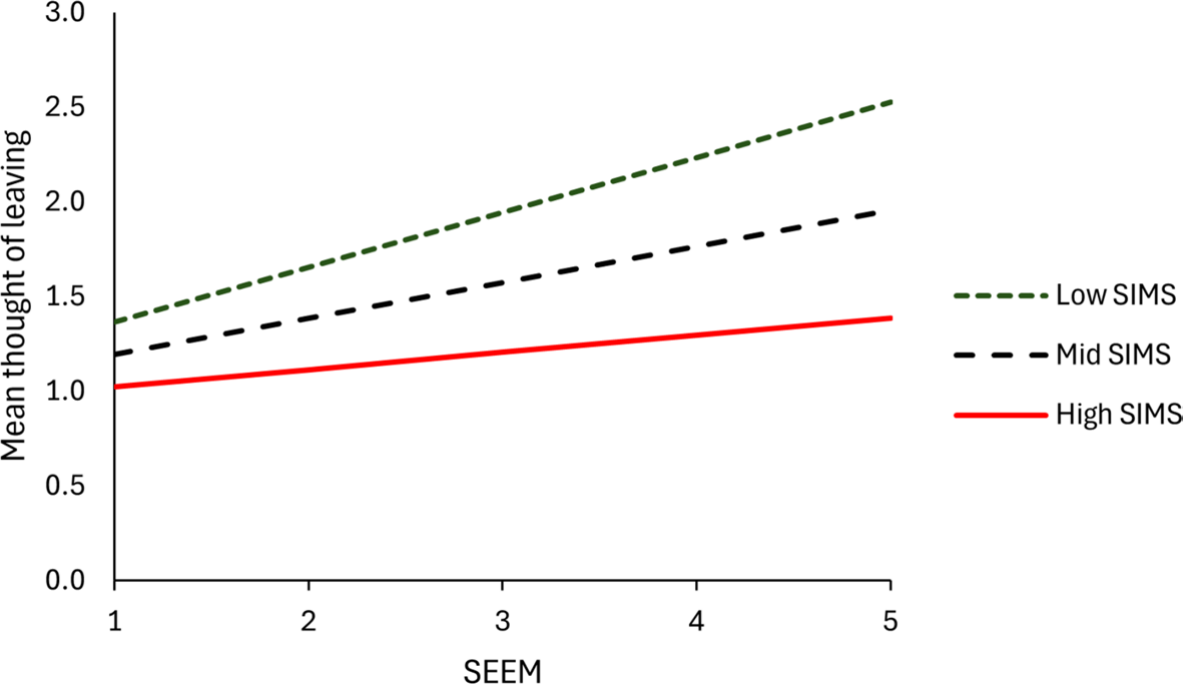
Interaction of SEEM and SIMS on thoughts of leaving ministry

The fourth step in data analysis (see Table 5) explores the correlations between the two scales proposed by the Francis Burnout Inventory Revised (SEEM and SIMS) and age, years in ministry, thoughts of leaving ministry, and scores recorded on the Self-Compassion Scale proposed by Neff (2003a, 2003b). The correlation between SEEM and SIMS was − .56. The data presented in Table 5 demonstrate three main points. First, in this sample both age (*r* = − .22, *p* < .001) and years ministry (*r* = − .14, *p* < .05) impact scores recorded on the scale of emotional exhaustion in ministry. When both age and years in ministry were regressed on emotional exhaustion age remained significant, but years in ministry no longer remained significant. Younger clergy experience higher levels of emotional exhaustion. This is consistent with the consensus of previous research in the field and may be explained either as an age effect (suggesting that older clergy have learnt how to deal more effectively with the causes of emotional exhaustion) or as a cohort effect (suggesting that among the older cohorts those priests particularly susceptible to emotional exhaustion have already exited the priesthood). On the other hand, in this sample age did not impact scores recorded on the scale of satisfaction in ministry. This difference in the effect of age on the measures of positive affect and negative affect lend further support to the theory underpinning the balanced affect model, namely that positive affect and negative affect operate as partially independent systems. Second, in this sample, thoughts of leaving ministry are associated with higher levels of emotional exhaustion in ministry *and* with lower levels of satisfaction in ministry. This finding is consistent with previous research in the field. Third, in this sample high self-compassion scores are associated with lower levels of emotional exhaustion in ministry *and* with higher levels of satisfaction in ministry. This finding is consistent with the findings of Barnard and Curry (2012) and Fabri et al. (under review), both of whom also tested the association between scores recorded on the scale of self-compassion proposed by Neff (2003a, 2003b) and the two scales proposed by the Francis Burnout Inventory (see Table 6 and Fig. 1).

**Table 5:** Correlations with Scale of Emotional Exhaustion in Ministry and Satisfaction in Ministry Scale

	SEEM *r*	SIMS *r*
Age	− .22^***^	.10
Years in ministry	− .14^*^	.04
Thoughts of leaving ministry	.48^***^	− .44^***^
Self-compassion	− .60^***^	.56^***^

^*^ *p* < .05; ^***^ *p* < .001

**Table 6:** Regression models: Thoughts of leaving ministry

	Model 1	Model 2	Model 3
*Personal factors*			
Age	− .14^*^	− .05	− .04
*Burnout scales*			
Scale of Emotional Exhaustion in Ministry		.32^***^	.32^***^
Satisfaction in Ministry Scale		− .25^***^	− .21^***^
*Interaction*			
SEEM x SIMS			− .14^*^
R^2^	.020	.272	.289
∆	.020^*^	.252^***^	.018^*^

Predictors were mean centred and the table reports standardised beta weights. ^*^p < .05; ^***^p < .001

The fifth step in data analysis employs multiple regression to test the theory of balanced affect and does so in three steps, regressing the independent variables on thoughts of leaving ministry. In these models the continuous variables have been mean centred. In model 1 age is introduced into the equation. These data demonstrate that younger clergy are more likely than older clergy to entertain thoughts of leaving ministry. After taking age into account, model 2 introduces both emotional exhaustion in ministry and satisfaction in ministry into the equation. These data confirm that positive affect and negative affect both contribute independent and cumulative effects on thoughts of leaving ministry. Higher scores of emotional exhaustion are associated with more frequent thoughts of leaving ministry; while, higher scores of satisfaction in ministry are associated with less frequent thoughts of leaving ministry. Model 3 introduces the interaction term between satisfaction in ministry and emotional exhaustion in ministry. The term is negative, indicating that the positive correlation between thoughts of leaving ministry and emotional exhaustion is greater among those with low satisfaction than among those with high satisfaction, as illustrated in Fig. 1. This supports the notion that affect balance exists between emotional exhaustion in ministry and satisfaction in ministry in terms of their effect on clergy burnout.

### Limitations

The main limitation with the present study is that it relied on a snowball sample of willing participants. While this is an appropriate sample on which to test the psychometric properties of the instrument, it may be inflating the reported levels of positive affect and depressing the reported levels of negative affect. Further research is needed that can build a more systematic study of Catholic priests in Portugal.

## Conclusion

The aim of the present study was to translate the Francis Burnout Inventory Revised into Portuguese and to test the application of this translation among a sample of Catholic priests serving in Portugal. This aim was achieved by drawing on data provided by a snowball sample of 266 Catholic priests (91% diocesan and 9% religious). Four conclusions can be drawn from analyses of these data.

First, both scales proposed by the Francis Burnout Inventory Revised (SEEM and SIMS) reported a high level of internal consistency reliability, and with an alpha coefficient of .89. Second, both scales confirmed previous established findings against two measures hypothesised as relating with SEEM and SIMS in opposite directions: thoughts of leaving ministry and scores recorded on the Self-Compassion Scale proposed by Neff (2003a, 2003b). Third, the theory that positive affect and negative affect operate as partially independent systems was supported by the finding that; while, scores of emotional exhaustion declined significantly with age, scores of satisfaction in ministry were unrelated to age. Fourth, the finding that positive affect (SIMS) reduced the effect of negative affect (thoughts of leaving ministry) support the balanced affect approach to conceptualising burnout among priests. Moreover, the additional effect of the interaction term between SEEM and SIMS on reducing thoughts of leaving ministry confirms that the mitigating effects of positive affect on the debilitating effect of negative affect increases with increasing levels of negative affect. Together these four conclusions confirm that the Portuguese translation of the Francis Burnout Inventory Revised is performing in ways commensurate with the parent English-language version of the instrument. On these grounds, the translation can be commended for use in further research.

As well as testing the Portuguese translation of the Francis Burnout Inventory Revised among Catholic priests serving in Portugal, data from the present study can contribute new insights into the work-related psychological wellbeing of these priests. Two further conclusions can, therefore, be drawn from the data. First, in terms of emotional exhaustion in ministry, up to a third of the priests were showing some signs of burnout, with 34% reporting that fatigue and irritation were part of their daily experience. Second, in terms of satisfaction in ministry, the majority of priests were showing signs of contentment, with 92% reporting that they have accomplished many worthwhile things in their current ministry. The good news behind these data is that positive affect remains high. The less good news is that this high level of positive affect is masking a significant level of negative affect.

## Data Availability

Data are available from the corresponding author upon reasonable request.
